# Acronyms and Opportunities for Improving Deep Nets

**DOI:** 10.3389/frai.2021.732381

**Published:** 2021-12-13

**Authors:** Kenneth Church, Boxiang Liu

**Affiliations:** Baidu Research, Sunnyvale, CA, United States

**Keywords:** acronyms, multiword expressions, Ab3P, ERNIE, BERT, deep nets

## Abstract

Recently, several studies have reported promising results with BERT-like methods on acronym tasks. In this study, we find an older rule-based program, Ab3P, not only performs better, but error analysis suggests why. There is a well-known spelling convention in acronyms where each letter in the short form (SF) refers to “salient” letters in the long form (LF). The error analysis uses decision trees and logistic regression to show that there is an opportunity for many pre-trained models (BERT, T5, BioBert, BART, ERNIE) to take advantage of this spelling convention.

## 1 Introduction

Deep net models such as BERT (Devlin et al. (2019)), GPT (Brown et al. (2020)), ELMo (Peters et al. (2018)), ERNIE (Sun et al. (2020)), T5 (Raffel et al. (2020)), BioBert (Lee et al. (2020)), BART (Lewis et al. (2020)) have achieved record-setting performance on a wide range of tasks. However, there are always opportunities for improvement.

It is standard practice in end-to-end machine learning to emphasize certain types of evidence and de-emphasize other types of evidence. So too, in formal linguistics, following the discussion of performance and competence in Chomsky (1965), there has been a tradition to emphasize certain types of evidence, e.g., linguistic competence, sound and meaning, and de-emphasize other types of evidence: linguistic performance, corpus statistics, orthography. Acronyms pose a challenge for these practices in machine learning and linguistics since orthography plays an important role in acronyms. There is a well-known spelling convention in acronyms where each letter in the short form (SF) refers to “salient” letters in the long form (LF). We will compare deep nets with a rule-based system in §7, and find that the rule-based system produces better F-scores because it takes advantage of orthography. Error analysis in §8.1 will show that many of the errors in BERT, BART, ERNIE, T5 and BioBERT involve orthography.

Acronyms are a special case of multiword expressions (MWEs) (Krovetz et al. (2011)). It is common in certain types of technical writing to abbreviate compounds (and MWEs) with acronyms. The first mention of an acronym in a document is likely to be a definition, where both the SF and LF are introduced in a way that makes it clear that the SF will be used in subsequent mentions to refer to the LF. As we will see in §3.2, some acronym tasks in the literature take advantage of definitions, though some do not.

## 2 What do we Mean by Multiword Expressions?

Multiword expressions include many topics. Some treatments of MWEs distinguish idioms from collocations and technical terminology (Barkema (1996)), though many of the linguistic tests mentioned below apply to idioms as well as collocations, terminology and many other linguistic phenomena.

Baldwin and Kim (2010) provide the following examples of MWEs:^1^

San Francisco, ad hoc, by and large, Where Eagles Dare, kick the bucket, part of speech, in step, the Oakland Raiders, trip the light fantastic, telephone box, call (someone) up, take a walk, do a number on (someone), take (unfair) advantage (of), pull strings, kindle excitement, fresh air

**FIGURE 1:**
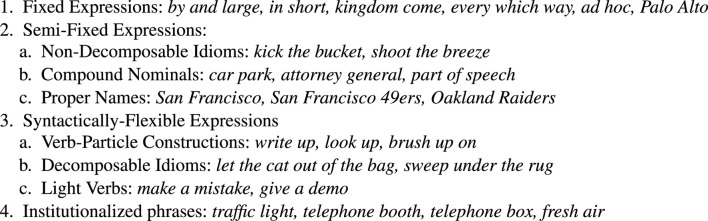
A taxonomy of Multiword Expressions (MWEs) from Sag et al. (2002).

**FIGURE 2:**
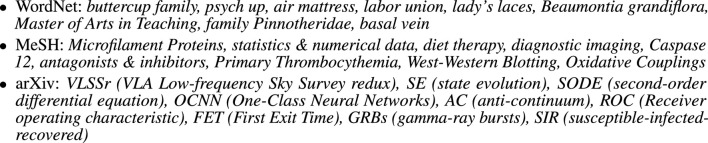
Examples of Multiword Expressions (MWEs) from WordNet, MeSH and arXiv.

Some treatments are closer to linguistics and some are closer to engineering. It is interesting to compare the linguistic treatment in Figure 1 with the engineering treatment in Figure 2. The taxonomy in Figure 1 emphasizes linguistically interesting constructions such as idioms and verbs, unlike the examples in Figure 2, where there are more nouns phrases (collocations and technical terminology). Idioms and verbs are central to many fascinating linguistic puzzles. Noun phrases are more common, especially in corpora based on technical documents.

Linguistic resources contain many instances of MWEs. Sag et al. (2002) estimate that 41% or more of the entries in WordNet 1.7 (Feldbaum (1998)) involve MWEs. MWEs are even more frequent in technical writing such as medical documents; 46% of the entries in MeSH contain spaces. MeSH^2^ (Medical Subject Headings) is a controlled vocabulary thesaurus from NLM (National Library of Medicine). 32 million abstracts can be downloaded from PubMed^3^ with MeSH annotations.

Linguistic discussions of MWEs often make use of linguistic tests such as productivity and compositionality. Idioms tend to have relatively fixed word order, and resist substitution. Semantics tend to be non-compositional; in general, the meaning of an idiom cannot be derived from the meaning of the parts.

Distributional statistics also tend to be non-compositional. That is, the frequency of the whole is not what one would expect from the frequency of the parts. In other words, idioms tend to have large pointwise mutual information (PMI) (Church and Hanks (1990)), where the probability of the whole is larger than chance (based on the parts). Goldberg and Levy (2014) established a connection between PMI and more modern methods such as static embeddings (Mikolov et al. (2013)). Static embeddings are similar to deep nets such as BERT, though those methods are referred to as contextual embeddings to emphasize the fact that they capture more contextual constraints than static embeddings. Both static and contextual embeddings are taking advantage of Firth (1957): “you shall know a word by the company it keeps.”

§8.3 will introduce *freq*, a frequency-based feature that will be used in error analysis to identify additional opportunities for improvement. Acronyms for *herpes simplex virus* and *artificial intelligence*, for example, are defined in thousands of documents. Since so many acronyms are defined in so many documents, there are opportunities to take advantage of constraints across documents in addition to constraints within documents.

## 3 Acronym Tasks

### 3.1 Tasks

At least four acronym tasks have been proposed in the research literature: ADI, SQuAD, AI and AD.

**FIGURE 3:**
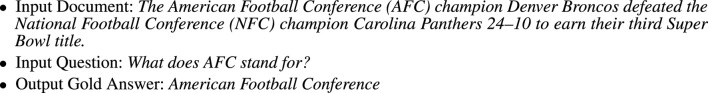
An example of the SQuAD (Stanford Question Answering Dataset) Task. The gold answer is a span, a substring from the input document.

**FIGURE 4:**
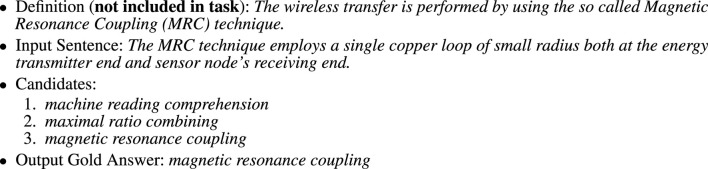
An example of the AD (Acronym Disambiguation) Task. The system is asked to return one of the candidates. This task would be easier (and more realistic) with more context (including definitions). Input sentences were selected from arXiv. This example comes from https://arxiv.org/pdf/1805.07795.pdf, where the definition appears immediately before the input sentence.

1) The ADI (Abbreviation Definition Identification) (Sohn et al. (2008)) task takes one or more texts as input, and finds pairs of short forms (SFs) and long forms (LFs) that are defined in the input texts. There are no restrictions on the length of the input texts.
2) SQuAD (Stanford Question Answering Dataset) (Rajpurkar et al. (2016)) takes a question and a short document (containing no more than 512 subword tokens) as input, and returns an answer, where the answer is a span (substring) from the input document. Many ( ∼1% ) of the 100,000 SQuAD questions involve acronyms, as shown in Figure 3.
3) AI (acronym identification)^4^ (Pouran Ben Veyseh et al., 2020) takes a sentence as input. The input sentence may contain SFs and/or LFs. The task is similar to NER (named entity recognition). Following Ramshaw and Marcus (1995), the NER task takes a text as input and tags each input word with **B**, **I** or **O**, where **B** tags begin a span (named entity), **I** tags are inside a span and **O** tags are outside a span. The AI task replaces the 3 BIO tags with 5 tags: **B-short**, **B-long**, **I-short**, **I-long** and **O**, where **B-short** and **I-short** are for SFs, and **B-long** and **I-long** are for LFs.
4) AD (acronym disambiguation)^5^ takes a sentence as input. The input sentence contains an ambiguous SF. The task is to choose the appropriate LF from a set of candidates, as illustrated in Figure 4. The AD task is intended to be similar to WSD (word-sense disambiguation) (Navigli (2009)).

The ADI task was proposed well before the other tasks, and remains, in our opinion, a more realistic task for practical applications. There are no constraints on the length of the input documents for ADI, unlike the other tasks where the input cannot exceed 512 subwords.

### 3.2 D Tasks vs. D-Less Tasks

D-tasks (ADI and SQuAD) take advantage of definitions (D), and D-less tasks (AI and AD) do not. This paper will focus on D-tasks, which are easier (and more useful) than D-less tasks because definitions establish clear connections between SFs and LFs.

Definitions would be less common if they were unhelpful. When authors make the effort to write definitions, we should pay attention to what they have written. This paper will focus on definitions, and will not attempt to make sense of acronyms taken out of context, in contrast to D-less tasks, where definitions are not included in the inputs to the system.

Consider the example in Figure 4. Definitions provide a clear connection between SFs and LFs. Without definitions, SFs would be much more ambiguous. The SF, *MRC*, for example, can refer to a number of different LFs: *machine reading comprehension, maximal ratio combining, magnetic resonance coupling*. Many LF expansions are possible in other documents, but definitions make it clear which LF is intended in this document. In this case, the missing definition appears immediately before the input sentence; see https://arxiv.org/pdf/1805.07795.pdf for the larger context. The missing definition is also presented in Figure 4, but labeled as not included in the task.

If we take advantage of the definition, then all we need is simple string matching because the SF and the LF appear next to one another in the definition. Without the definition, the disambiguation task becomes much more challenging. It may well require so-called “AI complete” knowledge to connect the dots between *copper loops* and *magnetic resonance coupling*.

## 4 Long-Distance Dependencies

Since the distance between the definition and subsequent mentions is typically more than 512 subwords, we either need a D-less mechanism for processing subsequent mentions, or a mechanism for capturing long-distance dependencies (Chomsky (1956; 1957)) between definitions and subsequent mentions. There are many well-known mechanisms for capturing these dependencies such as finite-state automata (FSA) and recurrent neural networks (RNNs).

On the other hand, there are also good reasons to limit information flows. Transformers such as BERT limit information flows for reasons that are somewhat analogous to REST APIs. Representational state transfer (REST) is a widely accepted set of guidelines for creating stateless, reliable program interfaces on the web; much of the popularity and success of REST APIs can be attributed to the absence of state. State (memory) is very powerful, which is both a blessing as well as a curse. APIs with state can do more than APIs without state, but the industry prefers simpler and more reliable APIs over more powerful APIs in many use cases. So too, nets that can capture long-distance dependencies can do more than nets that cannot capture long-distance dependencies, but there are trade-offs between power and other considerations such as simplicity and reliability.

If one wants to limit information flows, there are a couple of ways to capture long-distance dependencies. One approach involves multiple passes. For example, in footnote 12, we refer to a dictionary of tuples <docId,SF,LF> that we extracted from a large collection of arXiv documents. This can be thought of as a global memory that can pass information over long distances between definitions and subsequent mentions. These tuples can pass information both within and across documents. §8.3 will introduce *freq*, a feature which takes advantage of frequencies of SFs and LFs aggregated over many documents. §8.4 will show that *freq* can be used to improve BERT’s performance.

Alternatively, we could increase the size of the context, though there are a number of practical concerns. GPU memory is expensive and memory requirements grow quadratically with the length of the input sequence because of attention mechanisms in most BERT-like methods. That said, there are reasons to hope it might become more feasible to relax the 512 limitation, given recent progress such as: Dai et al. (2019); Zaheer et al. (2020); Kitaev et al. (2020); Wang et al. (2020); Katharopoulos et al. (2020); Beltagy et al. (2020).

## 5 Ab3P

Long inputs (more than 512 subword units) are not a problem for older ADI systems developed more than a decade ago. Some of these older systems use rules (Schwartz and Hearst (2002)) and others (Kuo et al. (2009)) use machine learning methods that predate transformers. One of the more effective solutions is Ab3P (Abbreviation Plus Pseudo-Precision)^6^ (Sohn et al. (2008)).

Note that Ab3P is both the name of a program as well as the name of a benchmark (a dataset plus gold labels for SF-LF pairs). This confusion is admittedly unfortunate, but it reflects the fact that Sohn et al. (2008) contributed in two important ways that have stood up well to the test of time. To avoid confusion, we will use “Ab3P system” and “Ab3P benchmark” to distinguish the system from the benchmark.

The Ab3P system takes one or more texts as input and uses a set of 17 manually-created rules to capture patterns such as: Alpha Beta (AB), Alpha**-**Beta (AB), Alpha Beta**s** (ABs), Alpha of the Beta (AB). Rules like these can be implemented with FSAs, making it possible to extract definitions and SF-LF pairs from arbitrarily long inputs. These rules focus on definitions, as opposed to D-less tasks (AI and AD) that attempt to connect the dots between SFs and LFs without the benefit of definitions.

It is hard to compare D-tasks with D-less tasks, but two of the four tasks in §3.1 are D-tasks, so they can be compared with one another. In §7, we will compare Ab3P system with BERT-SQuAD. Given all the excitement over recent advances in deep nets (Bengio et al. (2021)), we were surprised to discover that deep nets for SQuAD do not work as well as old-fashioned rules. In addition to performance on standard benchmarks, the Ab3P systems has a number of additional advantages that are important in practical applications: speed, memory, cost, ease of use.

Practical applications such as Pubtator Central^7^ (Wei et al. (2012, 2013, 2019); Leaman and Lu (2016)) are currently using the Ab3P system. Pubtator is a web service for viewing and retrieving annotations of PubMed documents. The Pubtator service also provides links for downloading tens of millions of abstracts with annotations for bioconcepts^8^ Full articles with annotations are available for about 10% of the collection.

### 5.1 Standard Benchmarks for ADI Systems

Most evaluations of ADI systems use four standard benchmarks based on PubMed, as shown in Table 1: Ab3P (Sohn et al. (2008)), BIOADI (Kuo et al. (2009)), MEDSTRACT (Wren et al. (2005)) and SH (Schwartz and Hearst (2002)). These benchmarks include input texts, typically PubMed abstracts, as well as gold labels for SFs and LFs. The benchmarks do not specify how they were selected from PubMed, though they appear to cover a broad set of topics in biology, chemistry and medicine. The SF-LF pairs in the test sets are defined in one of the sentences in the input text. Table 1 reports the length of these definitions in characters. These benchmarks are available for download in XML format, without markup^9^.

**TABLE 1:** Four standard benchmarks for ADI evaluations.

Benchmark	Corpus size	SF-LF pairs	Abstract (characters)
Ab3P benchmark	1250 PubMed Abstracts	1223	1378 ± 494
BIOADI	1200 PubMed Abstracts	1720	1456 ± 398
MEDSTRACT	199 PubMed Citations	159	1126 ± 411
SH	1000 PubMed Abstracts	979	1407 ± 464

### 5.2 Acronyms From arXiv

There have been concerns that methods developed for these benchmarks may not transfer well to other domains such as arXiv papers. To address these concerns, we ran the Ab3P system on 1.65M arXiv articles (with LATEXmarkup)^10^.

The Ab3P system was probably not designed for LATEX input. We could have stripped the markup, though given the lack of a clear consensus on how to strip markup,^11^ we decided that it was simpler to see how well Ab3P would work without stripping markup. We were pleasantly surprised to discover that markup can be helpful, at least in certain cases, such as the use of italics in definitions. Of course, there are other cases where markup confuses Ab3P, such as equations.

Since we believe the arXiv acronyms will be useful to readers interested in this topic, results are posted on GitHub^12^ (with some filtering). After filtering, Ab3P found 8.3M SF-LF pairs (an average of 5.0 pairs per article).

How well does Ab3P work on arXiv articles? Since we do not have a test set for arXiv articles, we extracted a sample of 210 SF-LF pairs from the Ab3P output described above (before filtering). Two judges labeled each pair as either good 1) or bad (0). Judgments are posted on GitHub (see footnote 12) under the eval subdirectory. Results in Table 2 suggest that Ab3P agrees with the judges almost as much as the judges agree with one another, at least after filtering.

**TABLE 2:** Agreement on a sample of 210 SF-LF pairs from arXiv extracted with the Ab3P system. After filtering, there are 161 pairs. See footnote 12 for these judgments, as well as 8.3M SF-LF pairs.

Comparison	Filtered (%)	Unfiltered (%)
Judge 1 vs. Judge 2	96	95
Judge 1 vs. Ab3P system	94	77
Judge 2 vs. Ab3P system	96	80

The filter was simply a regular expression that required SFs to contain at least two upper case letters. This filter was designed to remove difficult cases that the judges were likely to disagree on, as well as noise in Ab3P output such as fragments of mathematical expressions in LATEX. This filter removed 49 of 210 SFs. Of the 49, both judges agreed that 36 were bad (0), and 10 were good (1).^13^ The judges disagreed on the remaining 3 SFs.

Precision is easier to evaluate than recall. Table 2 shows promising precision, but says little about recall. In other words, it is easier to estimate errors of commission than errors of omission on a collection that is too large to annotate exhaustively.

It is standard practice to report recall based on standard benchmarks that are small enough to annotate exhaustively. Although we will follow that standard practice in Table 3, we are concerned that estimates of recall based on standard benchmarks may be inflated since the community has been working with these benchmarks for years/decades. There is too much opportunity to tune for these benchmarks.

**TABLE 3:** Ab3P has better F-scores on four standard benchmarks based on PubMed.

**Benchmark**	**Ab3P System**	**BERT-SQuAD**
Ab3P Benchmark	**0.889**	0.794
BIOADI	**0.838**	0.698
MEDSTRACT	**0.943**	0.844
SH	**0.858**	0.769

## 6 Related Work

As mentioned above, the ADI task is similar to other tasks such as: SQuAD, AI and AD. There is a considerable body of work on all these tasks, though the literature on SQuAD^14^ is more extensive than the literature on AI and AD because the AI and AD tasks are newer. AI and AD were introduced at a recent AAAI-2021 workshop, SDU@AAAI-2021^15^ (Jaber and Martínez (2021)). Deep nets such as BERT-SQuAD^16^ are the method of choice in the question-answering (Q&A) literature these days. Most of the papers at SDU@AAAI-2021 also used deep nets.

As mentioned above, the AD task is similar to word-sense disambiguation (WSD) and the AI task is similar to named entity recognition (NER). Surveys on WSD (Navigli (2009)) and NER (Nadeau and Sekine (2007)) cover much of the literature before deep nets.

NER is used for a number of tasks that extract spans, substrings of input texts, and label spans with tags. ACE^17^ used tags such as person, organization, location, etc., (Doddington et al. (2004)). Many benchmarks use BIO tags to label spans. Each word in the input text is tagged as B (begins a span), I (inside a span) or O (otherwise). Some benchmarks introduce tags such as B-disease and B-chemical to distinguish disease entities from chemical entities. Lee et al. (2020) show BioBERT, a version of BERT trained on PubMed abstracts, is effective on a number of such NER benchmarks:^18^.

1) NCBI disease (Doğan et al. (2014)).
2) i2b2/VA (Uzuner et al. (2011)).
3) BC5CDR (Li et al. (2016)).
4) BC4CHEMD (Krallinger et al. (2015)).
5) BC2GM (Smith et al. (2008)).
6) JNLPBA (Kim et al. (2004)).
7) LINNAEUS (Gerner et al. (2010)).
8) Species-800 (Pafilis et al. (2013)).

Much of the work on these benchmarks has been incorporated into PubTator (see footnote 8). PubTator identifies NER spans and tags them with six bioconcepts: genes, diseases, chemicals, mutations, species and cell lines. PubTator also links entities to ontologies such as MeSH. PubTator links the gene p53, for example, to different points in the ontology for different species: humans, mice, fruit flies, etc. Pubtator makes it easy to download millions of abstracts and papers with these annotations.

There have been concerns that work based on PubMed may not generalize well to other domains. For this reason, the AI and AD benchmarks at SDU@AAAI were based on arXiv documents, as opposed to PubMed documents.

As mentioned above, this paper will focus on D tasks (ADI, SQuAD) as opposed to D-less tasks (AI and AD). D-less tasks attempt to address difficult problems that are similar to WSD and NER, but there is no need to address these difficult problems because definitions and larger contexts make the task much easier, as discussed in Figure 4. In that example, there is a very useful definition, but it was not included in the inputs to the system, even though the definition appears immediately before the sentence that was provided as input to the system.

## 7 BERT-SQuAD: An Alternative to Ab3P for ADI

The task of finding LFs can be reduced to a Q&A task by converting SFs to questions of the form: *What does* < **SF** > *stand for?* If the document is a definition, then BERT-SQuAD will often return the LF.

Five short ( ∼30 lines) SQuAD programs for BERT, BART, BioBERT, ERNIE and T5 have been posted on GitHub.^19^ The code has been simplified as much as possible, to make it easy to see what it is doing, and what it is not doing. These programs read SFs and definitions from standard input, and output LFs to standard output.

Note that this task is somewhat easier than ADI where the input is an arbitrarily long text, and the program is not only expected to find LFs, but also definitions and SFs. For the comparisons in Table 3, we give BERT-SQuAD a few unfair hints from the Ab3P system. In particular, we use Ab3P to find the SF and the sentence containing the relevant definition.^20^ Even with these unfair hints, BERT-SQuAD is less effective than Ab3P, as shown in Table 3.

## 8 Error Analysis

Error analysis shows that many of the errors are “off-by-one.” That is, LF candidates from BERT-SQuAD tend to contain one word too many or one word too few, as shown in Tables 4, 5.^21^.

**TABLE 4:** Many LF candidates from BERT-SQuAD are off by one word.

Benchmark	Correct (%)	Off-by-one (%)	Otherwise (%)
Ab3P Benchmark	87	10	3
BIOADI	83	11	6
MEDSTRACT	88	8	3
SH	80	14	6

**TABLE 5:** Some examples of BERT-SQuAD errors.

SF	LF Candidate	Context
DL	limen	This study examined and compared bilabial compression force difference limen (DL) values …
HSV	Latent herpes simplex virus	Latent herpes simplex virus (HSV) has been demonstrated …
HC	controls	…in healthy controls (HC) T cells …
PDT	bedside percutaneous dilational tracheostomy	…who were treated with bedside percutaneous dilational tracheostomy (PDT) because of …
PD	transepithelial potential difference	…by means of transepithelial potential difference (PD),…
CK	Total creatine kinase	Total creatine kinase (CK) and CK-B activity in …
EC	human embryonal carcinoma	…pluripotent human embryonal carcinoma (EC) cells,…
PI	Serine proteinase inhibitor	Serine proteinase inhibitor (PI)-9 with a …
RPMS	perceived muscle soreness	…and rating of perceived muscle soreness (RPMS) on five consecutive mornings
AVMs	cerebral arteriovenous malformations	Fifty-one patients with 59 angiographically proven cerebral arteriovenous malformations (AVMs) were examined by …
hAR	androgen receptor	The human androgen receptor (hAR) is a …

It is common for definitions to parenthesize either the LF or the SF:

1) AB (Alpha Beta)
2) Alpha Beta (AB)

There are relatively few errors in the first case because the LF is delimited on both sides by parentheses. In the second case, the right edge of the LF is relatively easy because the LF is delimited by a parenthesis between the LF and the SF. The crux is to determine the left edge of the LF. Most of the “off-by-one” errors involve the left edge of the second case.

### 8.1 Charmatch

What is BERT-SQuAD missing? As suggested above, it appears that BERT is failing to take advantage of the crucial spelling convention. Consider the example from Table 5: healthy ***c*** ontrols (HC). In this case, BERT-SQuAD drops the first word from the LF, returning *controls* instead of *healthy controls*. BERT’s candidate violates the spelling convention where the characters in the SF should match the salient (***red***) characters in the LF. It is relatively easy for the Ab3P system to capture this spelling convention with rules such as regular expressions. A quick inspection of the BERT-SQuAD program in footnote 19, however, suggests that BERT-SQuAD does not attempt to capture this spelling convention. It ought to be possible, of course, for deep nets to capture the convention with a combination of token-based and character-based nets.

The point of this paper is not so much to improve performance; the Ab3P system is already doing very well, with performance close to inter-annotator agreement, as suggested in Table 2. This paper is more concerned with setting appropriate expectations. Deep nets often outperform traditional methods, but not always. Traditional methods are likely to do well when there is an obvious rule like the spelling convention that does not fit neatly into the deep net framework.

We hypothesize that BERT-SQuAD is missing the spelling constraint. To test this hypothesis, we introduce a simple Boolean feature, *charmatch*, that compares the first character of the SF to the first character of the candidate LF. Table 6 shows that candidates from BERT-SQuAD are more likely to be correct when these characters match, confirming the hypothesis.

**TABLE 6:** BERT-SQuAD does not capture *charmatch*.

Benchmark	Pr (*correct charmatch* = 0)	Pr (*correct charmatch* = 1)
Ab3P Benchmark	0.15	0.96
BIOADI	0.07	0.94
MEDSTRACT	0.18	0.98
SH	0.10	0.94

This charmatch feature is easier to implement than the more general spelling constraint involving salient characters, since it can be tricky to define what counts as “salient.” Given the crux mentioned above, the first character is helpful for identifying the left edge of the LF, and the left edge addresses the bulk of the opportunity.

There are, of course, a few examples where the charmatch heuristic is unhelpful. The first character of the LF is not always the same as the first character of the SF in a few exceptions such as “chloride current (ICl)” and “transepithelial resistance (Rt).”

### 8.2 Using Decision Trees in Error Analysis

**FIGURE 5:**
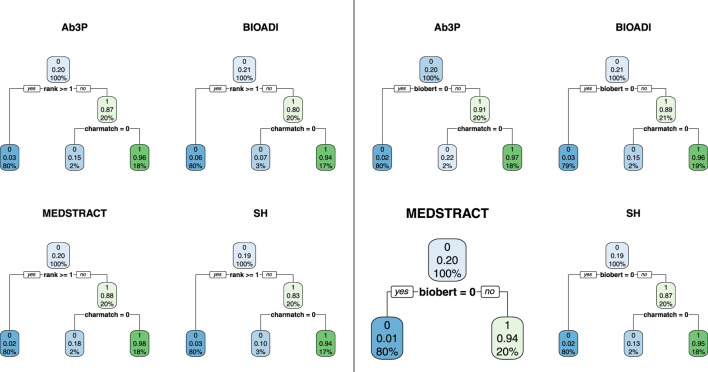
All but one of these decision trees split on *charmatch*, suggesting that charmatch is bringing new information not captured by the first split. There are 8 trees (4 benchmarks and 2 equations). The four trees on the left use Eq. 1 and the four on the right use Eq. 2.

**FIGURE 6:**
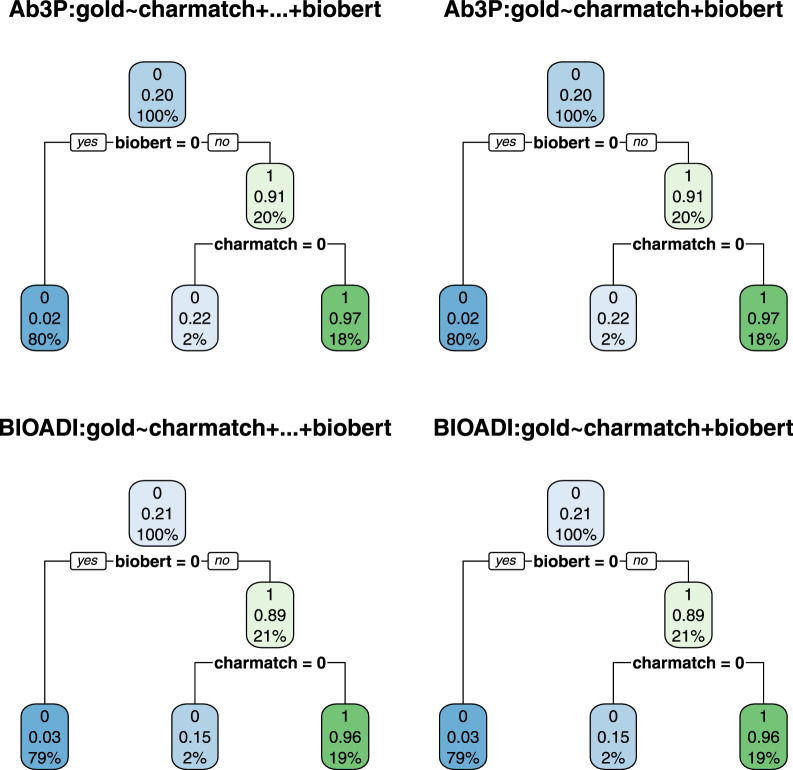
The decision trees on the left are based on Eq. 3 whereas the trees on the right are based on Eq. 2. The fact that the trees on the left are the same as the trees on the right suggests that 2 features (*charmatch* and *biobert*) are as useful as all 7 (*rank, charmatch, bert, bart, ernie, t5* and *biobert*).

Figures 5, 6 use decision trees to establish the usefulness of *charmatch*. Note that all but one of the trees split on *charmatch*, suggesting that *charmatch* offers important information that is not already captured by deep nets such as BioBERT, BERT, BART, ERNIE and T5.

These trees were created with the *rpart* package^22^ in R. Rpart takes a data table and an equation as input, and outputs a decision tree. Figures 5, 6 are based on four data tables and three Eqs 1–3.

gold∼rank+charmatch (1)

gold∼rank+charmatch+biobert (2)

gold∼rank+charmatch+bert+bart+ernie+t5+biobert (3)

The data tables are constructed as follows. For each benchmark (Ab3P, BIOADI, MESTRACT and SH), use the Ab3P system to extract SFs and sentences containing definitions. Run BERT-SQuAD to find 5 candidate LFs for each SF. The 5 candidates are assigned a *rank* between 0 (top position) and 4.

Each candidate is also assigned seven binary features:

• *gold*: 1 iff the LF candidate is correct
• *charmatch*: 1 iff the first character of the SF is the same as the first character of the candidate LF (ignoring upper and lower case).
• *bert, bart, ernie, t5, biobert*: 1 iff the candidate LF was found by *bert, bart, ernie, t5, biobert*, respectively (ignoring white space).

The plots report three numbers for each node: 1) best label, 2) accuracy of best label and 3) coverage. Coverage values sum to 100% at each level in the tree. For example, at the root, there is a single node with 100% coverage (by construction). At the next level, there are two daughters. Again, by construction, since the training data has 5 candidates, and one of them is usually correct, then the gold set consists of about 20% 1s and 80% 0s. As a result, the first split usually covers 20% of the input with a label of 1, and 80% with a label of 0.

The accuracy of this first split, however, depends on the deep net in question. Compare the left four trees with the right four trees in Figure 5. Note that the accuracy of the first split is considerably better in the right panel, because BioBert is better than BERT. When the decision tree has access to the *biobert* feature (right four trees), then it will split on that, and ignore *rank* from BERT. Otherwise (left four trees), the first split is on the top choice from BERT (*rank* 0).

In Figure 5, the split at the next level is on *charmatch*. As mentioned above, the splits on *charmatch* suggest that *charmatch* is bringing new information to the table that is not already provided by the deep nets.

Figure 6 provides additional evidence in favor of *biobert* and *charmatch*. The trees on the left are the same as the trees on the right, but the trees on the left were given seven input features (Eq. 3), whereas the trees on the right were given just three input features (Eq. 2). Under a decision tree framework, the 2 input features, *biobert* and *charmatch* are more useful than the other 5: *rank, bert, bart, t5, ernie*.

To summarize this subsection, decision trees make a strong case for the value of *charmatch*. Decision trees are easy to interpret, and work well for binary features like *charmatch*. Other methods such as logistic regression may be more effective for error analysis with gradient features such as *rank* and *freq*. Decision trees could have introduced splits between the second best candidate (*rank* 1) and third best candidate (*rank* 2), but none of the trees did that. While the discussion of decision trees in this subsection failed to show a significant difference between the second best candidate and the third best candidate, §8.4 will use a logistic regression framework to make that distinction. The logistic regressions will also make use of an additional feature, *freq*.

### 8.3 Freq

In addition to *charmatch*, we identified another promising feature, *freq*, that takes advantage of constraints across documents. Consider the example from Table 5: *Latent herpes simplex virus (HSV) has been demonstrated in …* As mentioned in Table 4, BERT-SQuAD is often off by one. In this case, the candidate LF is one word too long: *Latent herpes simplex virus*. This example is relatively easy because both the *charmatch* and *freq* features point in the correct direction.

The *freq* feature takes advantage of the fact that many of these SFs are defined in thousands of PubMed abstracts. The *freq* feature uses suffix arrays (Manber and Myers (1993)) to count the number of matches of: LF + ‘(’ + SF in PubMed. In this example, we found 6075 instances of “herpes simplex virus (HSV”, but only 8 instances of “Latent herpes simplex virus (HSV.” Of course, raw frequencies need to be normalized appropriately because shorter strings tend to be more frequent than longer strings.

### 8.4 12 Models: Rank + Charmatch + Freq

This section adds logistic regression to the error analysis to avoid difficult normalization and feature combination questions. Table 7 established plenty of opportunities for reranking to improve performance. The decision trees in §8.2 make it clear that top position (*rank* 0) is more likely to be correct than other positions, but Table 7 makes the stronger statement: candidates with smaller *ranks* are more likely to be correct than candidates with larger *ranks*. Decision trees showed that the top candidate is better than the second candidate, but they did not show that the second best candidate is better than the third best candidate. We will use logistic regressions to make the stronger statement.

**TABLE 7:** # correct by rank (position in n-best list). The top choice (rank 0) is often correct, but there is plenty of room for improvement since there are correct candidates in ranks 1–4.

Rank	Benchmark			
	Ab3P Benchmark	BIOADI	MESTRACT	SH
0	920	1129	128	698
1	85	103	11	58
2	19	124	3	25
3	8	68		4
4	6	33		

This section introduces a dozen logistic regression models to rerank the top 5 candidates from BERT. As shown in Table 8, models 1–4 use Eq. 4, models 5–8 use Eq. 5 and models 9–12 use Eq. 6. *y* comes from the four gold sets: models 1,5 and 9 use the Ab3P benchmark for *y*, models 2, 6 and 10 use BIOADI, models 3, 7 and 11 use MEDSTRACT, and models 4, 8 and 12 use SH.

y∼rank (4)

y∼rank+charmatch (5)

y∼rank+charmatch+log(1+freq) (6)

**TABLE 8:** Twelve regression models: 1–12. The gold labels, *y*, depend on the columns, and the number of input features, *x*, depends on the rows.

Equation	Ab3P benchmark	BIOADI	MEDSTRACT	SH
Eq. 4	1	2	3	4
Eq. 5	5	6	7	8
Eq. 6	9	10	11	12

Coefficients are shown in Table 9. All coefficients are significant. Reranking sorts candidates by *z*, as defined in Table 9. Pr (*correct*) ≈ *σ*(*z*) where *σ*(*z*) = 1/(1 + *e* ^−*z*^).

**TABLE 9:** Twelve logistic regression models: *z* = *β*_0_ + *β*_1_ *rank* + *β*_2_ *charmatch* + *β*_3_   *log* (1 + *freq*).

Coef	Model											
	1	2	3	4	5	6	7	8	9	10	11	12
*β*_0_	1.6	0.7	1.9	1.4	−1.2	−2.5	−1.0	−1.9	−2.7	−5.2	−3.2	−3.1
*β*_1_	−3.3	−1.6	−3.9	−3.3	−3.2	−1.5	−4.0	−3.2	−2.9	−1.5	−3.8	−2.9
*β*_2_	0	0	0	0	3.5	3.8	3.9	4.1	3.7	5.2	4.7	4.3
*β*_3_	0	0	0	0	0	0	0	0	0.3	0.5	0.4	0.3

One of the advantages of simple models like regression is interpretability. The fact that all the coefficients are significant means that all three features are useful. If BERT-SQuAD was already taking advantage of these opportunities, then *β*_2_ and *β*_3_ should not be significant, and performance for models 5–12 should be no better than models 1–4.

**FIGURE 7:**
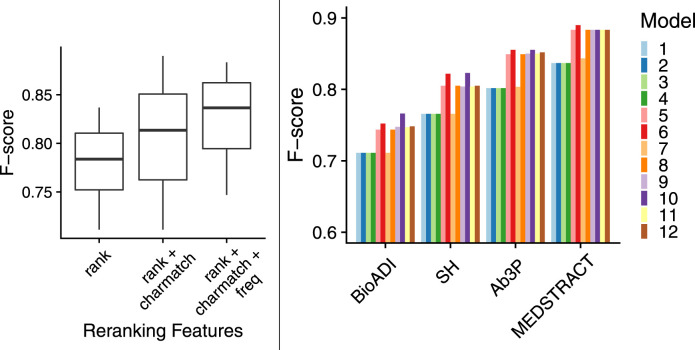
More features → better F-scores; boxplots (left panel) summarize 48 bars (right panel).

The boxplots in Figure 7 show that models with more features are better than models with fewer features. That is, both *charmatch* and *freq* are contributing additional information that is not being captured already by BERT-SQuAD. The boxplots in the left panel summarize 48 bars in the right panel. The 48 bars cover all combinations of the 12 models and the four benchmarks. The boxplots make it easier to see contributions from *charmatch* and *freq*, whereas the 48 bars are complicated by other factors that might be distracting such as the fact that some benchmarks are easier than others.

The fact that *β*_1_ < 0 in all 12 models suggests that less is more, at least for *rank*. That is, candidates are more likely to be correct if they have smaller ranks (near the top of n-best list from BERT-SQuAD). Recall that decision trees in §8.2 were able to show the top candidate was better than other candidates, but they were not able to make the strong statement about candidates in other positions.

The fact that *β*_2_ > 0 and *β*_3_ > 0 suggests that more is more for *charmatch* and *freq*. That is, candidates are more likely to be correct if they have more of these features. The best candidates will pass the *charmatch* test, and they will be defined together in many other documents.

Table 10 shows that models with more features are more confident. Models with more features are not only more likely to be correct, but the log likelihood scores are larger when they are correct. This is additional evidence that BERT-SQuAD is not capturing *charmatch* (spelling conventions) and *freq* (constraints across documents).

**TABLE 10:** Models with more features are more confident when they are correct. Models 1–4 use a single feature (*rank*). Models 5-8 add *charmatch*. Models 9–12 add freq. Confidence is estimated as median *σ*(*z*) for correct candidates.

Features	Ab3P benchmark	BIOADI	MEDSTRACT	SH
1 (Models 1–4)	0.829	0.660	0.865	0.796
2 (Models 5–8)	0.908	0.787	0.945	0.901
3 (Models 9–12)	0.936	0.948	0.975	0.949

## 9 Discussion and Future Work

### 9.1 Document-Level Context: First Mention *vs*. Subsequent Mentions

It is common in linguistics to distinguish first mentions from subsequent mentions. For acronyms, the first mention is often a definition that connects the dots between a SF and a LF. These definitions enable subsequent mentions to be shortened to the SF, avoiding unnecessary repetitions of the LF.

Acronyms can be viewed as a special case of references that span well beyond the level of a sentence. There has been considerable discussion of given/new information (Chafe (1974); Clark and Clark (1977); Prince (1981); Terken and Hirschberg (1994)) where the first mention (new information) tends to be accented (emphasized when spoken), unlike subsequent mentions (given information).

The distinction between first mentions and subsequent mentions is also important for coreference (Hobbs (1979); Elsner and Charniak (2008); Baumann and Riester (2013)), where subsequent mentions tend to be shorter (and less emphasized) than first mentions. Subsequent mentions are often just a pronoun. It is common to refer to the first mention as *marked* and subsequent mentions as *unmarked*. In news articles, for example, the first mention of a person is likely to include a full name along with a role such as “spokesman” unlike subsequent mentions which are reduced to a surname or a pronoun.

From a statistical point of view, the first mention of a concept tends to be more surprising (less likely), but once an unusual term has been put on the table, it is much more likely to be mentioned again. Church (2000) reported that Noriega was not likely to be mentioned in the AP news. Only 0.78% of AP articles mentioned Noriega, even in 1990 when the US invaded his country (Panama). But if one of those articles mentioned him once, it is very likely that he will be mentioned again.

In general, for almost all words, and especially for content words and good keywords for information retrieval (such as acronyms), first mentions are more surprising (less likely) than subsequent mentions (Church and Gale (1995); Katz (1996)):

$${\mathrm{Pr}}_{w}\left(k\geq 1\right)\ll {\mathrm{Pr}}_{w}\left(k\geq 2|k\geq 1\right)$$ (7)

where *Pr*_*w*_(*k*) be the probability of seeing exactly *k* instances of a word *w* in a document. Thus, *Pr*_*w*_(*k* ≥ 1) is the probability that *w* will be mentioned in a document, and *Pr*_*w*_(*k* ≥ 2|*k* ≥ 1) is the probability that *w* will be mentioned again, given that it was mentioned at least once.

Under standard independence assumptions (such as a Poisson process), one would expect the first mention to be about equally likely as the second mention, but these assumptions ignore document-level contexts, which are important for tasks such as information retrieval (and perhaps acronyms). The difference between the first mention and subsequent mentions can be large, often a couple of orders of magnitude, especially for good search terms like Noriega. In the case of Noriega in 1990 AP news, the first mention, *Pr*_*w*_(*k* ≥ 1) ≈ 0.79*%*, is almost 100x smaller than subsequent mentions, *Pr*_*w*_(*k* ≥ 2|*k* ≥ 1) ≈ 71*%*.

These observations have consequences for acronyms. Good keywords tend to be repeated within documents. SFs are also likely to be repeated. If a definition introduces a SF, there is a strong presupposition that the SF will be used again later in the document. Otherwise, if the SF is not used again, the definition serves little purpose.

This repetition property could be used to avoid the filter in §5.2 for fragments of mathematical expressions in LATEX. Such fragments have distributions that are closer to Poisson, unlike SFs, which are more likely to be repeated.

### 9.2 Constraints Within and Across Documents

The experiments in this paper focus on definitions, though in future work, we would like to consider unusual documents that refer to acronyms without definitions. There may be some opportunities to take advantage of resources such as a dictionary of acronyms derived from arXiv documents (as described in footnote 12).

As mentioned above, acronyms tend to be defined before they are used, though there are a few exceptions for acronyms that are extremely well known such as RADAR, SONAR, PCR, PCA, AI. In some cases, acronyms may not be defined because the SF is more memorable and more meaningful than the LF. Some examples of memorable SFs involve the recent interest in Sesame Street: BERT (Devlin et al. (2019)), ERNIE (Sun et al. (2019)) and Elmo (Peters et al. (2018)).

Although acronyms are sometimes used in a paper without definition, it is often possible to find a definition in other papers. Thus, one can construct a dictionary of SFs and LFs, even for well-known acronyms, by running Ab3P on a large corpus of documents, and aggregating SF-LF pairs over many documents.

Consider the acronym, *AI*, which is often used without definition. However, there are many definitions of this acronym in the 1.65M arXiv articles mentioned in §5.2. Among the 8.3M SF-LF pairs extracted by Ab3P, there are 5243 pairs with LF expansions for **AI**. The most common LF expansion is *artificial intelligence*, not surprisingly, though there are a number of other possibilities including: *Anderson insulator, absorption index, asynchronous irregular, atom interferometer, autoionization* and many more. There are more than 4k expansions of *AI* as *artificial intelligence*, with some variation in the use of upper and lower case, as well as italics. In a few articles, the LF is in a language other than English.

Although a SF such as *AI* can refer to different LFs in different documents, it is unusual for a SF to be used in two different ways within the same document. This article is an exception, since we use *AI* to refer to both *Acronym Identification* as well as *Artificial Intelligence*. Most documents are not exceptional in this way, and obey a “one sense per discourse” constraint like word senses (Gale et al. (1992)), creating opportunities for systems that take advantage of larger document-level contexts that span well beyond sentences (and batches of 512 subword tokens).

## 10 Conclusion

This paper discussed methods for extracting short forms (SFs) and long forms (LFs) of acronyms, an interesting special case of Multiword Expressions (MWEs). Given all the recent excitement over deep nets, we were surprised to discover that older rule-based methods are still being used in practical applications such as Pubtator. In our experience, the Ab3P system is better than deep nets on the ADI task, not only in terms of precision and recall, but also in terms of speed, memory and ease of use. We ran the Ab3P system on 1.65M arXiv articles and posted 8.3M SF-LF pairs on GitHub.

We also posted 5 short ( ∼30 line) programs on GitHub for extracting LFs using pretrained models fine-tuned for SQuAD. The 5 programs are based on BERT, BART, ERNIE, T5 and BioBERT. These programs make it clear what deep nets are doing, and what they are not doing.

We are not advocating decision trees and reranking as a practical solution, but merely as a method for error analysis. The proposed reranking method addresses the question: what is BERT missing? Reranking identified at least two answers: spelling conventions (*charmatch*) and constraints across documents (*freq*).

For practical applications, there may not be much room for improvement over the Ab3P system. Ab3P is already doing very well on the four standard benchmarks in Table 1. We also reported promising results in Table 2, with precision close to inter-annotator agreement.

## Data Availability

The datasets presented in this study can be found in this repository: https://github.com/kwchurch/AB3P_arXiv. Example code for the AD task can be found in this repository: https://github.com/kwchurch/bert_acronym.py.

## Footnotes

1
https://people.eng.unimelb.edu.au/tbaldwin/pubs/altss2004.pdf

2
https://www.ncbi.nlm.nih.gov/mesh/

3
https://pubmed.ncbi.nlm.nih.gov/

4
https://github.com/amirveyseh/AAAI-21-SDU-shared-task-1-AI

5
https://github.com/amirveyseh/AAAI-21-SDU-shared-task-2-AD

6
https://github.com/ncbi-nlp/Ab3P

7
https://www.ncbi.nlm.nih.gov/research/pubtator/

8
https://ftp.ncbi.nlm.nih.gov/pub/lu/PubTatorCentral/

9
http://bioc.sourceforge.net/BioCresources.html

10
https://arxiv.org/help/bulk_data

11
https://tex.stackexchange.com/questions/252203/tex-to-plain-text-or-doc

12
https://github.com/kwchurch/AB3P_arXiv

13 The filter fails for a few exceptions where the SF contains no upper case letters: “quaternionic contact (qc),” “proton-deuteron (pd)” and “metric measure spaces (mm-spaces).”

14
https://paperswithcode.com/sota/question-answering-on-squad20

15
https://sites.google.com/view/sdu-aaai21/home

16
https://huggingface.co/bert-large-uncased-whole-word-masking-finetuned-squad

17
https://www.ldc.upenn.edu/collaborations/past-projects/ace

18
https://github.com/dmis-lab/biobert-pytorch/tree/master/named-entity-recognition

19
https://github.com/kwchurch/bert_acronym.py/blob/main/bert_acronym.py

20 Most of these sentences were short enough to meet the 512 subword limit, but a few sentences were too long. For this reason, we found it necessary to wrap the code to catch errors associated with long inputs. Some statistics on the lengths of definitions are reported in Table 1.

21 Note that F-scores in Table 3 should not be compared with %Correct in Table 4.

22
https://cran.r-project.org/web/packages/rpart/rpart.pdf
